# Accounting for False Positive HIV Tests: Is Visceral Leishmaniasis Responsible?

**DOI:** 10.1371/journal.pone.0132422

**Published:** 2015-07-10

**Authors:** Leslie Shanks, Koert Ritmeijer, Erwan Piriou, M. Ruby Siddiqui, Jarmila Kliescikova, Neil Pearce, Cono Ariti, Libsework Muluneh, Johnson Masiga, Almaz Abebe

**Affiliations:** 1 Médecins Sans Frontières, Amsterdam, The Netherlands; 2 Médecins Sans Frontières, London, United Kingdom; 3 London School of Hygiene and Tropical Medicine, London, United Kingdom; 4 Ethiopian Health and Nutrition Research Institute, Addis Ababa, Ethiopia; Instituto de Higiene e Medicina Tropical, PORTUGAL

## Abstract

**Background:**

Co-infection with HIV and visceral leishmaniasis is an important consideration in treatment of either disease in endemic areas. Diagnosis of HIV in resource-limited settings relies on rapid diagnostic tests used together in an algorithm. A limitation of the HIV diagnostic algorithm is that it is vulnerable to falsely positive reactions due to cross reactivity. It has been postulated that visceral leishmaniasis (VL) infection can increase this risk of false positive HIV results. This cross sectional study compared the risk of false positive HIV results in VL patients with non-VL individuals.

**Methodology/Principal Findings:**

Participants were recruited from 2 sites in Ethiopia. The Ethiopian algorithm of a tiebreaker using 3 rapid diagnostic tests (RDTs) was used to test for HIV. The gold standard test was the Western Blot, with indeterminate results resolved by PCR testing. Every RDT screen positive individual was included for testing with the gold standard along with 10% of all negatives. The final analysis included 89 VL and 405 non-VL patients. HIV prevalence was found to be 12.8% (47/ 367) in the VL group compared to 7.9% (200/2526) in the non-VL group. The RDT algorithm in the VL group yielded 47 positives, 4 false positives, and 38 negatives. The same algorithm for those without VL had 200 positives, 14 false positives, and 191 negatives. Specificity and positive predictive value for the group with VL was less than the non-VL group; however, the difference was not found to be significant (p = 0.52 and p = 0.76, respectively).

**Conclusion:**

The test algorithm yielded a high number of HIV false positive results. However, we were unable to demonstrate a significant difference between groups with and without VL disease. This suggests that the presence of endemic visceral leishmaniasis alone cannot account for the high number of false positive HIV results in our study.

## Background

Visceral Leishmaniasis is a vector-borne protozoan disease transmitted by the bite of the sandfly. Globally, it is estimated that 200,000–400,000 new cases of visceral leishmaniasis (VL) occur annually [1]. Ethiopia is one of the 6 highest burden countries, with the Tigray and Amhara regions particularly affected [1]. HIV infection is well known to interact with VL, and reported rates of HIV co-infection in Ethiopia are 20–40% [2].

HIV testing in resource-limited settings is done with a combination of rapid diagnostic tests (RDTs) used in a diagnostic algorithm [3]. The use of RDTs allows rapid scale up and wider coverage of HIV testing as the tests can be done outside of the laboratory and are low cost. However RDT algorithms are potentially vulnerable to false positive results due to cross reactivity. This risk has been documented in multiple countries with a variety of RDTs [4–9].

Médecins Sans Frontières-Holland (MSF) ran projects in Tigray and Amhara regions of Ethiopia to support the Regional Health Bureaus with diagnosing and treating VL. An additional programme component was HIV diagnosis and treatment. Testing was offered as voluntary counselling and testing (VCT), and as provider initiated diagnostic testing (PiCT) for ill patients. All VL patients were referred for HIV testing using rapid diagnostic tests (RDTs) prior to starting treatment. Since the introduction of HIV testing, patients and field staff identified concerns about misclassification of HIV patients. This risk was remarked to be higher amongst those with VL. For example, over a 16 month period in 2006–7 using a serial Determine/Unigold algorithm, the HIV-RDT discordancy rate was 4.7% (275 / 5879), whereas in VL patients it was 15.6% (83 / 532), (OR 3.77; 95% CI 2.87–4.95; p<0.001). Discordant results, where one RDT is positive and the other negative, are associated with a higher risk of false positive reactions as they represent underlying cross-reactivity. A cohort of falsely diagnosed HIV patients was identified retrospectively from the period of 2007–8 using confirmation testing. All had been initially diagnosed positive on the serial Determine/Unigold algorithm. Four of the 7 individuals had VL, however there was no comparison group [10]. The concern about the high individual and programmatic impact of false diagnosis of HIV led us to design a study evaluating the diagnostic algorithms used in Ethiopia, which is reported separately [11]. We included a secondary objective to compare the risk of false positive HIV diagnosis among patients with and without VL, which is the focus of this analysis.

## Methods

### Design

The design chosen was that of a cross-sectional study comparing two independent groups of individuals undergoing HIV testing, those with active VL disease and those without.

### Setting

The study was conducted in 2 sites in northwestern Ethiopia, a MSF-supported health centre in Abdurafi, Amhara Region and a hospital in Humera, Tigray Region. The populations included residents as well as high numbers of migrant workers who are present seasonally. Testing took place in the designated counselling and testing (CT) centres in each site, in addition to the antenatal clinic, hospital, and outpatient department in Humera. VL is endemic in both locations and treatment programmes exist in both sites specifically to address VL

### Test Algorithm

In Ethiopia, a tiebreaker regimen consisting of 3 RDTs in series is the national diagnostic algorithm. HIV (1+2) Antibody Colloidal Gold (KHB, Shanghai Kehua Bio-engineering Co Ltd, China) is used as a screening test, followed by HIV 1/2 STAT-PAK (Chembio Diagnostics, USA) if positive. Where the result of STAT-PAK is discordant with KHB, a third test, Unigold HIV (Trinity Biotech, Ireland), is used as a tiebreaker to determine the final result.

### Inclusion and exclusion criteria

All clients, aged > = 5 years, presenting to be tested for HIV in the study sites were invited to participate in the study. Participants were excluded if it proved impossible to obtain a venous blood sample.

### Sample size

A sample size of 90 VL patients was chosen based on a sample size calculation to detect a difference in positive predictive value (PPV) of 99% vs 90% in the non-VL and VL groups respectively with 80% power at the 95% significance level. To achieve this sample, all KHB positive samples were included along with every Nth KHB negative sample until the sample size was reached.

### Testing

Initial testing was done at the Counselling and Testing (CT) centres. All KHB positive results and 10% of the negatives were repeated in the laboratory. Laboratory technicians, blinded to the CT results, re- tested each sample on plasma with all 3 RDTs. Tests were performed according to manufacturer’s instructions and invalid tests discarded and repeated on new test devices.

All samples underwent testing by Western Blot (WB) with technicians blinded to earlier results using MP Diagnostics HIV Blot 2.2. Interpretation of results was based on American Red Cross recommendations [12].

Samples indeterminate on WB were repeated and if still indeterminate, underwent DNA PCR examination on dry blood spots (DBS). The final gold standard result was that of the Western Blot, and where Western Blot was indeterminate, the PCR result. Further details on the testing are reported elsewhere [11].

VL was diagnosed by physicians using a diagnostic algorithm incorporating clinical signs and laboratory confirmation [13].

### Quality Control

All staff underwent training on the study standard operating procedures by the MSF laboratory supervisor, and received regular monitoring and supervision.

### Analysis

Data was collected into an excel database designed for the study. The field coordinator was trained on using the database. Predictive values and sensitivity and specificity were estimated from the 2 x 2 table of observed results after weighting based on the sampling proportion of the KHB positive and negative samples. Confidence intervals for each of the test parameters were calculated using exact binomial intervals. Logistic regression was used to compare the performance of algorithms between the VL groups. Fisher’s exact test was used when logistic regression failed.

Statistical analysis was done using Stata version 12 (StataCorp, Texas, USA).

### Ethical Review

The study received approval from the MSF Ethics Review Board, the EHNRI Research and Ethical Clearance Committee, and the National Research Ethics Review Committee, Ministry of Science and Technology in Ethiopia. A study amendment was obtained to increase the initial sample of the main study, in order to achieve sufficient power for the VL objective.

All study participants underwent informed consent procedures and had a written consent form signed by the participant or the guardian.

## Results

**Fig 1:**
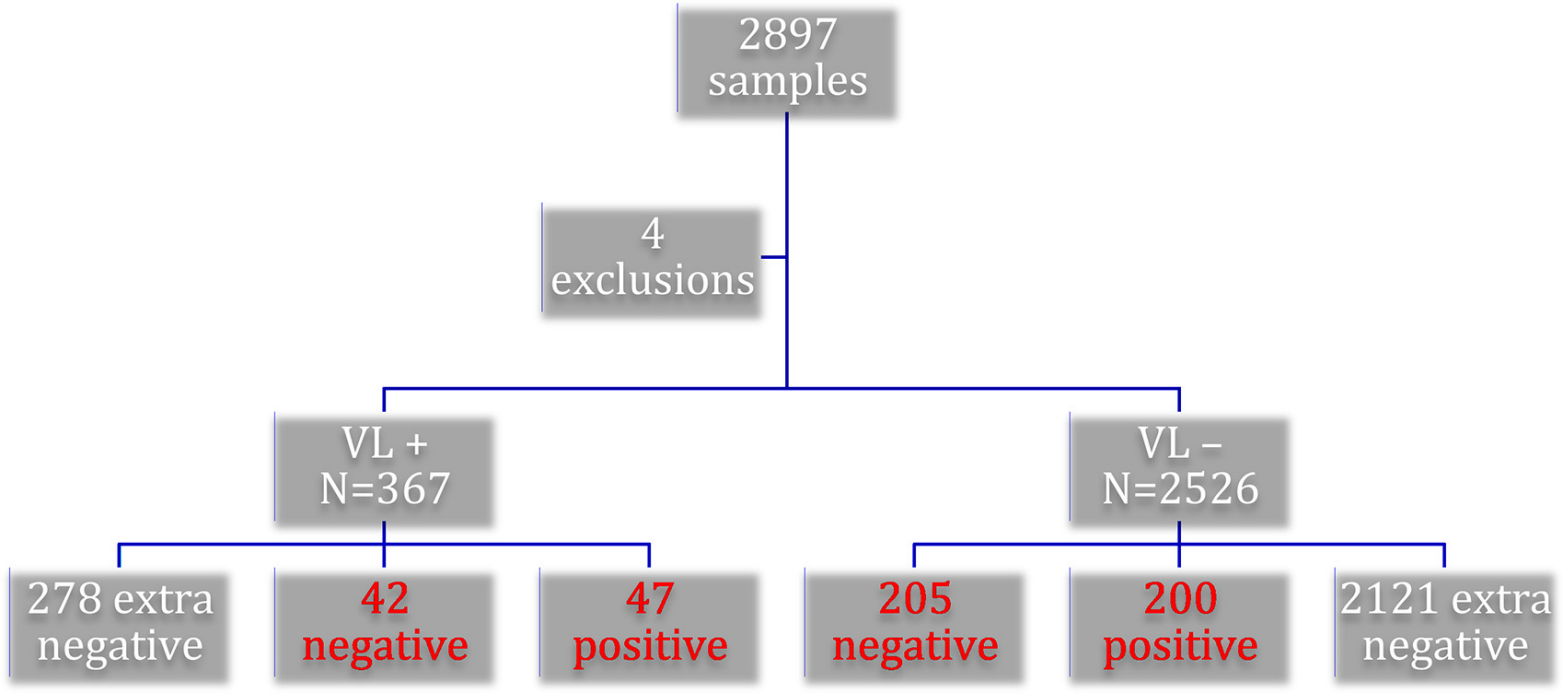
Overview of samples included in final analysis by visceral leishmaniasis (VL) status: Text boxes in red font indicate those samples confirmed by gold standard.

2897 participants were recruited to the study. Every KHB positive sample was tested with the gold standard, along with every 10^th^ KHB negative sample. Four samples were excluded: one duplicate, one without WB/PCR results, and two without VL status recorded. One patient was removed from the VL group as not meeting the diagnostic criteria for VL, leaving 89 VL and 405 non-VL patients for the final analysis as detailed in Fig 1.

Demographics of the sample are shown in Table 1.

**Table 1:** Demographics of study participants by visceral leishmaniasis (VL) status.

		VL (N = 89)	Non-VL (N = 405)	Total (N = 494)
**Age**	**Mean [range] in years**	28.9[12–63]	30.0 [10–67]	29.8 [10–67]
**Sex**	**Male**	86 (96.6%)	238 (58.8%)	324 (65.6%)
**Study Site**	**Abdurafi**	87 (97.8%)	178 (44.0%)	265 (53.6%)
**Residential status**	**Resident**	26 (29.2%)	215 (53.1%)	241 (48.8%)
	**Migrant**	55 (61.8%)	105 (25.9%)	160 (32.4%)
	**Settler**	8 (9.0%)	26 (6.4%)	34 (6.9%)
	**Other**	0	59 (14.6%)	59 (12.0%)
**Referral Site**	**Self-referral**	1 (1.1%)	234 (57.8%)	235 (47.6%)
	**In-patient**	13 (14.6%)	23 (5.7%)	36 (7.2%)
	**VL PiCT** *	60 (67.4%)	6 (1.5%)	66 (13.4%)
	**OPD** **	13 (14.6%)	121(29.9%)	134 (27.1%)
	**ANC** ***	0	21 (5.2%)	21 (4.3%)
	**Other**	2 (2.3%)	0	2 (0.4%)

*Provider initiated Counseling and Testing

**Outpatient Department

***Antenatal Care

HIV prevalence was found to be 12.8% (47/367) in the VL group compared to 7.9% (200/2526) in the non-VL group.

The RDT algorithm in the VL group had 47 positive, 4 false positives, and 38 negatives.

The same algorithm for those without VL had 200 positives, 14 false positives, and 191 negatives.

The VL group had a specificity of 99.1% compared with 99.4% in the non-VL group. The difference was not found to be statistically significant (p = 0.52). Similarly, while PPV was lower in the VL positive group (92.2% versus 93.5%), this difference was not found to be statistically significant (p = 0.76). Table 2 gives further details of the performance of the algorithm.

**Table 2:** Algorithm performance characteristics by visceral leishmaniasis (VL) status.

	Results (95% Confidence interval)			
	Sensitivity (95% CI*)	Specificity (95% CI)	Positive predictive value (95% CI)	Negative predictive value (95% CI)
**VL (N = 367)**	100% (92.5–100)	99.1% (97.7–99.8)	92.2% (81.1–97.8)	100% (90.8–100)
**Non-VL (N = 2526)**	100% (98.2–100)	99.4% (98.9–99.7)	93.5% (89.3–96.4)	100% (98.1–100)

* Confidence Interval

Individual RDT specificities reported by VL status are shown in Table 3.

**Table 3:** Specificity and positive predictive values of individual rapid diagnosis tests (RDTs) by visceral leishmaniasis (VL) status.

	Results (95% Confidence interval)	
	Specificity	PPV
**VL (N = 367)**		
KHB	98.5% (96.4–99.5)	90.4% (79.0–96.8)
Unigold	97.7% (95.4–99.1)	87.0% (75.1–94.6)
STAT-PAK	99.7% (98.2–100)	97.9% (88.9–99.9)
**Non-VL (N = 2526)**		
KHB	99.1% (98.7–99.5)	90.9% (86.3–94.4)
Unigold	99.2% (98.7–99.5)	91.2% (86.7–94.6)
STAT-PAK	99.9% (99.7–100)	99.0% (96.5–99.9)

## Discussion

Eighteen samples yielded false positive results using the Ethiopian national algorithm. The algorithm specificity in VL patients was worse than that in the cohort without VL. However this difference was not found to be statistically significant, suggesting that VL infection cannot account for the overall poor performance of the algorithm.

This is the first study that directly compares RDT algorithm performance in a VL-infected cohort with a non-infected cohort. There are two reports in the literature that suggest an association of increased risk of false positive HIV serological tests with VL. The first is a study from Brazil using HIV-1 ELISA as the screening test [14]. The authors report testing 100 samples positive for VL, of which 9 were falsely reactive for HIV on ELISA. The second paper is a case report whereby two 3^rd^ generation ELISA tests were falsely positive in a 32 year old man with VL [15].

There is evidence of cross-reactivity of HIV RDTs with another kinetoplastid protozoal disease Human African Trypanosomiasis (HAT) [16]. This study conducted in Democratic Republic of Congo tested 7 different RDTs at the time of diagnosis of HAT, and again 2 years after treatment completion. Specificities in the active disease group ranged from 39.1%-98.3%. Six of 7 RDTs improved in specificity in the treated patients; for 3 RDTs this change was statistically significant.

A proposed mechanism for false reactivity in VL and HAT is that of polyclonal B cell activation rather than a direct interaction with a VL or HAT antigen [14–17]. The immune response to visceral leishmaniasis involves polyclonal B cell activation as well as overproduction of interleukin-10 and hypergammaglobulinemia [18]. These responses are only normalized after successful treatment. However a number of other infections also cause polyclonal B cell activation. It may be that exposure to infectious agents common in Ethiopia cause a similar activation of the immune response, which would account for the lack of difference in VL patients. Here it is important to note that our comparison group included a mix of healthy individuals undergoing voluntary counselling and testing, as well as sick patients undergoing provider initiated counselling and testing. Non-specific cross-reactivity due to polyclonal B cell activation in response to a variety of infectious agents is consistent with previous reports that RDT specificity can be highly variable and difficult to predict in resource-limited settings [17,19]. The inability to predict which patients may have increased risk of cross reactivity to HIV tests, is an argument for the routine use of strong testing algorithms that include supplementary tests to confirm positive results.

A strength of this study is that we used a comparison group who had not been diagnosed with VL. We also controlled for seroconversion by resolving all indeterminate WB samples with PCR. A limitation is that we were unable to confirm all negative samples, and instead sampled a fixed proportion. We controlled for this in the analysis. We did not report on either length of infection prior to diagnosis of VL, nor did we separate VL cases on the basis of primary infection or relapse. We do not expect either of these factors to account for the lack of association found in this study however, as all cases of VL involved active disease and HIV testing occurred prior to treatment. Finally as we tested only 3 RDTs, we are unable to rule out that other RDTs may demonstrate a significant difference between VL infected and non infected groups as suggested by the high discordancy rates earlier observed with Determine and Unigold. Further studies would be needed to determine if there is an association with other RDTs and algorithm combinations.

## Conclusion

Despite several reports in the literature that visceral leishmaniasis is associated with an increased risk of false positive HIV results, we were unable to demonstrate a significant difference between groups with and without disease. This suggests that the presence of endemic visceral leishmaniasis alone cannot account for the high number of false positive HIV results in our study.
